# Use and Intentional Avoidance of Prescribed Medications in Pregnancy: A Cross-Sectional, Web-Based Study among 926 Women in Italy

**DOI:** 10.3390/ijerph17113830

**Published:** 2020-05-28

**Authors:** Angela Lupattelli, Marta Picinardi, Anna Cantarutti, Hedvig Nordeng

**Affiliations:** 1 PharmacoEpidemiology and Drug Safety Research Group, Department of Pharmacy, & PharmaTox Strategic Research Initiative, Faculty of Mathematics and Natural Sciences, University of Oslo, 0316 Oslo, Norway; marta.picinardi@gmail.com (M.P.); h.m.e.nordeng@farmasi.uio.no (H.N.); 2 Laboratory of Healthcare Research and Pharmacoepidemiology, Department of Statistics and Quantitative Methods, University of Milano-Bicocca, 20126 Milano, Italy; anna.cantarutti@unimib.it; 3 Division of Child Health, Norwegian Institute of Public Health, 0213 Oslo, Norway

**Keywords:** medication use, prescribed medication avoidance, pregnancy, Italy

## Abstract

Nation-wide information about medication use in pregnancy is lacking for Italy, and no study has so far investigated the prescribed medications which pregnant women deliberately avoid. In this study, we map medication use patterns in pregnancy, as well as the extent and type of prescribed medications which are purposely avoided by pregnant women in Italy. This is a sub-study within the “Multinational Medication Use in Pregnancy Study”—a cross-sectional, web-based study conducted in Italy from 7 November 2011 to 7 January 2012. Using an anonymous electronic questionnaire, we collected data from pregnant women and new mothers on medication use and deliberate avoidance during pregnancy and maternal characteristics. The sample included 926 women residing in Italy. The point prevalence of total medication use was 71.2%. Whereas 61.4% and 12.4% of women reported medication use for the treatment of short and longer-term illnesses, respectively, only 8.8% reported medication use for the treatment of both a short and a longer-term illness in pregnancy. We found no substantial differences in estimates across various geographical areas of Italy. Overall, 26.6% of women reported to have deliberately avoided a prescribed medication in pregnancy—most often nimesulide or ketoprofen, but also antibiotics. We conclude that prenatal exposure to medication is common among women in Italy, but estimates are lower than in other Western countries. Intentional avoidance of important medications by pregnant women raises concerns about the safeguarding of maternal–child health.

## 1. Introduction

Medication use in pregnancy has become an important public health concern in the last decades. Delayed childbearing—on the rise in most developed countries—and pregnant women’s preexisting disorders are among the factors posing greater risks of obstetrical complications [1,2]. Likewise, numerous acute or short-term illnesses—e.g., urinary tract infection (UTI) or nausea and vomiting—may negatively affect maternal–fetal health and well-being if sub-optimally treated [3,4]. For most of these disorders, whether short or longer-term, pharmacotherapy during pregnancy is often necessary.

Due to obvious ethical reasons, safety studies on medication in pregnancy cannot be conducted during embryogenesis in humans. Therefore, most medications are put on the market without establishing their safety profile in human pregnancy. To date, few medications have been shown to be major teratogens (e.g., warfarin, isotretinoin, valproate). However, the risk of minor teratogenicity or of more subtle effects on fetal and child development still has to be determined for most drugs [5,6,7]. Thus, understanding which—and to what extent—medications are taken in pregnancy has important clinical and public health implications [8].

Drug utilization research has shown that up to 80%–90% of women take at least one medication while pregnant, with variation in prevalence estimates across countries [9,10]. In Italy, region-specific studies [11,12,13] have found that between 48% and 70% of women are dispensed at least one conventional drug prescription in pregnancy. Including iron and folic acid, prescriptions were redeemed by 81% of pregnant women according a study conducted in Central Italy [13]. When based on maternal self-reporting, rates varied between 40%–63% [12,14,15]. However, the available literature is currently limited to regional contexts, and the last nation-wide investigation on the topic in Italy dates back to the end of the 1990s [14]. Because of the constant shift in the type and extent of exposure to medications during pregnancy, it is crucial to map nation-wide patterns of medication use in pregnancy, using data that are more recent [8]. This knowledge is crucial to prioritize medication safety research in pregnancy and to monitor maternal–child health at the population level.

At the same time, understanding which prescribed medications pregnant women deliberately avoid, despite their own need for treatment, is an important topic of research. Non-adherence to prescribed medication is a recognized problem, including among pregnant women [16]. If women are left without medical treatment, there may be important consequences for the health of both mother and child [17]. It is well-acknowledged that pregnant women perceive the risk of prescribed medications as unrealistically elevated, and treatments with nervous system medications are often discontinued [18,19,20,21]. However, it is currently unknown which medications pregnant women intentionally avoid in pregnancy, and which maternal factors are major predictors of such behavior.

The aim of this study was two-fold: (i) to examine the extent of and types of medications used during pregnancy in Italy, overall and for the treatment of short and longer-term illnesses; (ii) to map which prescribed medications are intentionally avoided by women during pregnancy and maternal factors associated with avoidance.

## 2. Materials and Methods

This is a sub-study within the “Multinational Medication Use in Pregnancy Study”—a cross-sectional, web-based study carried out in Europe, North and South America, and Australia to investigate patterns and correlates of medication use in pregnancy [10]. Pregnant women at any gestational age and new mothers of children under the age of 1 year were eligible for inclusion. In Italy, data were collected via an anonymous, self-administered electronic questionnaire (www.questback.com), accessible on-line between 7 November 2011 and 7 January 2012. The questionnaire was open to the public through banners posted on highly accessed pregnancy-related websites and fora (i.e., www.gravidanzaonline.it, www.forumsalute.it, www.mammole.it, www.pianetamamma.it, and www.miobambino.it), and social networks. The questionnaire was carefully designed to suit the internet administration approach. To improve the questionnaire completion rate, we applied specific technical features such as a multiple page design, routing of questions and a progress indicator of completion. Women answered the questions related to their current or latest pregnancy. Information about the internet penetration rate in Italy and the full questionnaire have been previously published [10]. To examine the study representativeness, we compared key characteristics of our study sample with those of the birthing population in Italy during the study period.

The study was piloted in Italy in September 2011 to ensure the comprehension and functionality of the electronic questionnaire and its suitability to the national context. The pilot study elicited no major change to the questionnaire. Data from the pilot were not included in the study dataset.

### 2.1. Maternal Factors

Pregnancy-related characteristics included the time of gestation or time since childbirth at the time of questionnaire completion, number of previous children, and use of folic acid before and/or during early pregnancy, as ascertained via maternal self-report. Maternal socio-demographic and life-style factors comprised age, attained educational level, occupation, marital status, smoking habits during pregnancy, and alcohol consumption after awareness of pregnancy. In addition, women were asked whether their mother tongue was different from Italian, which was considered as a proxy of immigrant status. Maternal correlates were categorized as presented in Table 1.

Women were presented with a list of short and longer-term illnesses and were asked to report whether they had experienced them during pregnancy. Short-term illnesses included nausea, constipation, heartburn or reflux problems, urinary tract infection, sleeping problems and pain conditions (i.e., pain in neck, back or pelvic girdle, and headache). Longer-term illnesses included somatic (i.e., asthma, allergy, epilepsy, diabetes I or II, cardiovascular and rheumatic diseases) and psychiatric (i.e., anxiety and depression) illnesses.

### 2.2. Medication Use and Avoidance

Women were asked standardized questions about medication use for specific short and longer-term illnesses, as described earlier [10]. For each indication, women could report the medications they took in pregnancy using free-text entry. It was optional to report the timing of exposure for each of the medication use questions (the alternatives were gestational weeks 0–12 (first trimester), 13–24 (second trimester) and 25-delivery (third trimester)). We defined a medication as a single product containing one or more active ingredients. We initially identified the main active ingredients and the formulation of the reported medicinal products either in the relevant national medicines database or textbook [22,23]. All recorded medications were coded into the corresponding Anatomical Therapeutic Chemical (ATC) codes at the ATC fifth level (i.e., the substance level) whenever possible; otherwise, they were coded into the second to fourth levels as appropriate [24]. We then quantified the extent and types of medications taken in pregnancy (yes/no variable), overall and by maternal illness (i.e., any short, any longer-term, or both a short and longer-term illness), and according to the timing of use in trimesters. The average number of medications taken during pregnancy was also calculated. Iron, mineral supplements, vitamins, herbal remedies and any type of alternative medicine were recorded separately and excluded from the estimation of medication use.

Intentional avoidance of prescribed medication during pregnancy was measured via the following question: “Have you deliberately chosen not to use a medicine prescribed by a doctor because you were pregnant?” In affirmative cases, women were asked to report the names of the avoided medications using free-text entry. Avoided medications were coded as described in the measurement of medication use above. The reason for the deliberate avoidance of prescription medicines was also captured using free-text entry. The study did not ask women about the timing in pregnancy in which the medication was avoided.

### 2.3. Ethics

Informed consent was given by the participants by ticking the answer “yes” to the question “Are you willing to participate in the study?” In Italy, the study was notified to the Ethics Board of the health district of Trento. All data were handled and stored anonymously. The Regional Ethics Committee in Norway, South-East region, granted an ethical approval exemption (2011/965 D) for the overall multinational study based on its anonymity.

### 2.4. Data Analysis

Descriptive statistics, corrected by survey weighting adjustment to make the results more representative of the birthing population in Italy, were used to characterize the prevalence of medication use and avoidance. The survey weight was based on the auxiliary variables of age and education, which are important correlates of study response [25]. National statistics based on Certificate of Delivery Assistance (CEDAP) data for 2012 provided information about the distribution of these variables among women who delivered in Italy [26]. Each woman was assigned a weight, obtained by dividing the population proportion by the corresponding sample proportion in each age-by-education strata [25]. Women under-represented in our sample (e.g., with lower education) were assigned a weight greater than one, while those over-represented (e.g., with higher education) received a weight smaller than one. In the attempt to quantify bias due to self-selection on the prevalence of medication use by trimester, both survey weighted and unweighted estimates were computed.

The odds ratios (ORs) of the intentional avoidance of prescribed medication (yes/no) with a 95% confidence interval (95% CI) were computed using univariate and multivariate logistic regression. Survey weighting adjustment was used to account for maternal age and education. Results are presented according to two levels of adjustment. The first analysis was an unadjusted, weighted logistic regression analysis. In the second analysis, we adjusted the model for selected covariates. The adjusted model was built as follows: first, candidate variables that included all the sociodemographic and life-style maternal characteristics shown in Table 1, longer-term somatic and psychiatric illnesses, and short-term UTI, nausea and pains, were selected based on a univariate *p*-value < 0.15. Longer-term illnesses were grouped into the somatic and psychiatric groups to avoid multicollinearity between the individual disorder variables, as many women reported comorbid longer-term disorders. This also applied to the various pain ailments measured in the study, which were therefore grouped together. Second, variables having no role (*p*-value > 0.05) or yielding a change smaller than 15% in the beta coefficients of the retained variables were removed from the model. Because there were few missing values for the study covariates (1.3%), these were handled by listwise deletion. The Hosmer and Lemeshow test was used to assess the goodness of fit of the final multivariate model [27]. In sensitivity analyses, we replicated the final multivariable model in pregnant and new mothers separately to address the risk of poor recall in the latter group. All statistical analyses were performed by using Stata version 16. Statistical significance was set at the 0.05 level.

## 3. Results

Of the 950 women who replied stating whether they were willing to participate in the study, 931 (98.0%) agreed to participate. We excluded five women because they were not residing in Italy at the time of the study, leaving a final study population of 926 women. Of these, the majority (*n* = 640, 69.1%) were pregnant at varying stages of gestation, mainly in the second trimester (first: 27.7%, second: 48.4%, and third trimester: 23.9%). The remainder (*n* = 286, 30.9%) were mothers who had delivered their children in the prior year, specifically between 0–6 months (*n* = 169, 59.1%) or 7–12 months (*n* = 117, 40.9%) prior to the completion of the study questionnaire. There was some variation in terms of the regional distribution of the responses (see Figure S1).

Table 1 outlines the maternal characteristics of the study population, overall and by medication use for short and longer-term illnesses. Table S1 shows the distribution of key indicators of our population versus the birthing population in Italy in the year 2012 (CEDAP statistics) [26]. The survey weight had a mean of 1.00 (sd: 0.96), whereas the median was 0.79 (interquartile range: 0.64–0.96); most women were thus down-represented in the analyses because of their higher educational level.

The point prevalence of ever using medication in pregnancy was 71.2% (95% CI: 67.1–74.9). A total of 552 women (61.4%, 95% CI: 57.1–65.5) reported use of medication for the treatment of short-term illnesses, whereas 135 did so for longer-term illnesses (12.4%, 95% CI: 10.1–15.2). A total of 98 women (8.8%, 95% CI: 6.9–11.2) reported use of medication for both short-term and longer-term illnesses during pregnancy. There was no substantial difference in medication use proportions between different geographical areas of Italy (see Figure S2). Generally, women with no previous children reported a lower extent of medication use to treat short-term illnesses than those with prior children. Women having pains or a UTI, or those who consumed alcohol in pregnancy, more often took short-term medication in pregnancy. Users of medication for longer-term illnesses were older and had a higher education level than non-users and had somatic and psychiatric longer-term illnesses in pregnancy more often. Users of medications for both short and longer-term illnesses had similar characteristics to women taking medication for longer-term illnesses in terms of their morbidity profile, education, and age.

On average, women took 1.7 medications (survey-weighted mean: 1.7, 95% CI: 1.6 to 1.9) during the course of the pregnancy, whereas the median number was one medication (interquartile range 0–2). There were nine women (1.2%) who reported the use of 8–11 medications in pregnancy; the majority reported either a single (25.4%), two (19.1%), or three or four (19.1%) medications. The remainder (6.5%) reported between five and seven medications in pregnancy. The most common combinations of medications taken in pregnancy were paracetamol with antacids (13.3%), paracetamol with antibiotics (11.7%), paracetamol with propulsive drugs (6.5%), and antacids with antibiotics (4.2%).

Table S2 shows the extent of overall medication use according to the trimester of pregnancy. Use of at least one medication ranged from 43.9% and 47.1% in the first and second trimester, respectively, then decreasing to 31.1% in the third trimester. The most common medication exposures in the first trimester included paracetamol, imidazole derivates, antacids or alginic acid, and levothyroxine. The use of antidepressants and anxiolytic benzodiazepines was higher in the first trimester (both 1.3%) compared to second or third trimesters (range 0.4–0.8%). Use of antacids and penicillins was greater in the second and third trimesters compared to the first trimester. As shown in Table S2, the survey-weighted prevalence estimates of medication use in the three trimesters did not largely differ from the unweighted estimates (range difference: 0–3.5% depending on the medication), although the 95% CI of the weighted results were broader.

Table 2 outlines the point prevalence of the most commonly reported medication exposures in pregnancy according to maternal illness. To treat short-term ailments, women most commonly took paracetamol (43.5%) during pregnancy, followed by medication for heartburn and gastric reflux (alginic acid: 9.8%), and antibiotics (broad spectrum penicillin: 7.5%; fosfomycin: 5.0%). Few women (*n* = 5) reported use of nimesulide or high-dose acetylsalicylic acid. In relation to longer-term illness, levothyroxine was the most used medication in pregnancy (5.1%), followed by anxiolytic benzodiazepines (1.4%), selective beta-2 agonists (1.4%), and selective serotonin re-uptake inhibitor (SSRI) antidepressants (1.1%). Almost 1% of women used antithrombotic agents; i.e., heparins and low-dose acetylsalicylic acid. Of the 98 women who reported medication use for the treatment of both short and longer-term illness, paracetamol (*n* = 68), levothyroxine (*n* = 48), alginic acid (*n* = 23) and penicillins with/without beta-lactamase inhibitors (*n* = 14) constituted the most common medications.

**Figure 1:**
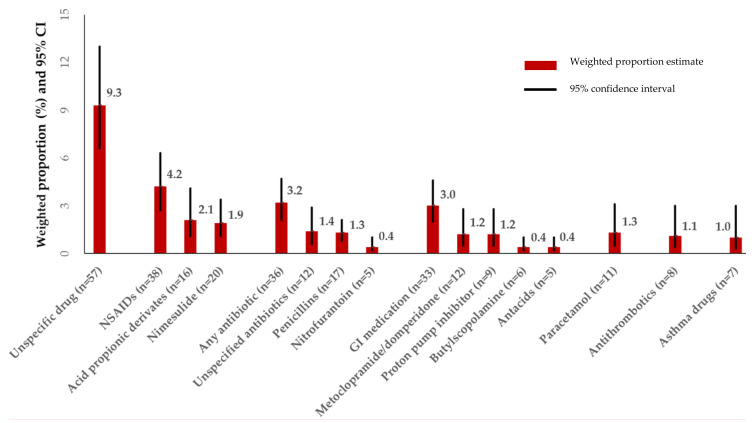
Most common medications intentionally avoided in pregnancy despite being prescribed (*n* = 926) ^1^. ^1^ Magnitude of proportions may not directly align with number of women in the various categories because proportions are weighted via survey weighting method. Abbreviations: 95% CI: 95% confidence interval; NSAID = Non-steroidal anti-inflammatory drugs; GI = Gastrointestinal.

Overall, 230 women (26.6%, 95% CI: 22.7–30.9) reported to have intentionally avoided one or more prescribed medications during pregnancy. As shown in Figure 1, many women (*n* = 57) did not report the name or group of the avoided medication. Non-steroidal anti-Inflammatory drugs (NSAIDs) (4.2%), mainly ketoprofen and nimesulide, antibiotics (3.2%) and gastrointestinal medications (3.0%), were the prescribed medications most often avoided. Of the women avoiding NSAIDs or antibiotics, 86.8% and 44.4% had ongoing pain ailments or UTI in pregnancy, respectively. About 1.1% of women did not take their prescribed heparins or low-dose acetylsalicylic acid, and 1.0% avoided their asthma medications. The most common reasons for medication avoidance were fear of fetal harm, contraindication in pregnancy in the drug label, and woman’s preference to cope with the illness rather than exposing the fetus to medication.

In the multivariate analysis, alcohol consumption after awareness of pregnancy, longer-term psychiatric illness in pregnancy, nausea or UTI during gestation were the sole variables significantly associated with intentional avoidance of prescribed medications in the final multivariate model. The corresponding measures of association are shown in Table 3. The results of this analysis conducted in the pregnant and new mother sub-samples did not meaningfully deviate from the main results (data not shown).

## 4. Discussion

This is the first internet-based, nation-wide study quantifying the use of medication in pregnancy among women in Italy. The study is also novel in providing insights into prescribed medications that are intentionally avoided during gestation and maternal factors related to this medication-taking behavior. We found that approximately seven out of ten women reported the use of at least one medication, either prescribed or over-the-counter, during the course of their pregnancy. Our point prevalence of medication use was 61.4% for treatment of short-term ailments, 12.4% for longer-term illnesses, and 8.8% for treatment of both, with no substantial differences across various geographical areas of Italy. One key finding is that approximately three out of ten women purposely avoided taking a prescribed medication in pregnancy— most commonly the NSAIDs ketoprofen or nimesulide, but also antibiotics. Of the vast array of sociodemographic factors examined, only alcohol consumption in pregnancy was associated with lower odds of medication avoidance in pregnancy, whereas multiple maternal illnesses were positively associated with this maternal behavior.

Our overall estimate of any medication use was fairly precise (71.2%, 95% CI: 67.1% to 74.9%) and generalizable to the target population of Italian women in terms of educational level and age, as we corrected our results for non-response to the study via survey-weighting methods. However, the observed total estimate for conventional medication use (excluding iron, supplements, and vitamins) was higher than that observed in two prior studies in the country (59.6% and 63.1%) [14,15]; different recruitment strategies—i.e., internet-based in our study versus outpatient gynecology and obstetrics clinics in the other studies—could partly explain this discrepancy. It is also possible that questions about medication exposures may be answered more truthfully in a web-based anonymous questionnaire than in a face-to-face interview [28], which could apparently inflate our point estimates compared to the abovementioned research [14,15].

Regarding the broader international perspective, our study confirms that prenatal exposure to conventional medication is lower in Italy than in other Western countries [9,29,30,31]. At the same time, our point prevalence is higher than those estimated in Asian countries (less than 20%), possibly due to contextual preferences towards complementary and alternative medicine and different clinical practice [32,33]. The systematic review by Daw et al. [9] showed that prescription drug use in pregnancy is highest in France (93%) and Germany (85%) and lowest in Northern European countries (44%–47%), even though most of the studies included in the systematic review used automated databases as a source of information about drug utilization, which are thereby limited to prescription-only medications. Data from the USA indicate that up to 94% of women use at least one medication in pregnancy, either prescribed or over-the-counter (OTC), with an average number of medications used as high as 4.3 (range 0–28) [31]. In the same US study, 50.1% of women took four or more medications by the end of 2008, and similar trends were observed in one additional study in a racial–ethnically diverse population of pregnant women across the USA [34]. Overall, these data suggest that the extent of polypharmacy in pregnancy is greater in the USA than in Italy, since in our study the average number of medications taken in pregnancy was substantially lower (mean 1.7, median 1.0), and only 8% of the women took between 5 and 11 medications. In line with prior studies in Europe and in Italy, we found that paracetamol was the most commonly used medication in pregnancy (45.3%) [15,35,36], largely for the treatment of acute and short-term illnesses of pregnancy. At the same time, few women (1.3%) rejected taking this analgesic in pregnancy despite being prescribed for pain conditions. This is somewhat surprising since paracetamol is generally regarded as a safe option in pregnancy in relation to the risk of congenital anomalies and other negative birth outcomes [17]. Furthermore, this study was conducted before concerns about the longer-term safety of paracetamol on child development start emerged in the literature [37]. However, as indicated in prior research, most women have a higher threshold for taking medication in pregnancy compared to when they are not pregnant, and they are more likely to refrain from medication use despite being ill [38]. Some women may prefer to suffer from pain than to expose their unborn child to any prescribed pharmacotherapy with analgesics.

After paracetamol, the most commonly used medications for short-term illnesses included drugs for acid-related disorders such as alginic acid, antacids and phloroglucinol, systemic antibiotics, mainly penicillins, fosfomycin and macrolides, nasal decongestants, and intra-vaginal imidazoles. Levothyroxine, anxiolytic-benzodiazepines, and inhaled selective beta-2 agonists were the most common medication exposures reported for the treatment of longer-term illness. These results are generally in line with previous research in Italy and other European countries [11,12,14,15,39], also in relation to less common drug exposures such as high-dose acetylsalicylic acid (0.5% in our study vs. 0.9% in Gagne et al. [11]) and nimesulide (0.3% vs. 0.4%, respectively). However, our estimates are somewhat lower than in the study by Ventura et al. [13], particularly in relation to antibiotics such as macrolides (1.7% in our study versus 27.6%) and broad spectrum penicillins (about 7.5% in our study versus 13.5%). This may be attributable, at least in part, to different data sources of medication exposure in the two studies; i.e., self-reported maternal use in our work versus redeemed prescription records in the other [13].

The NSAIDs—mainly represented by ketoprofen and nimesulide—constituted the prescribed medication group most commonly avoided by pregnant women (4.2%). Reluctance towards taking nimesulide as prescribed is not surprising given the paucity of safety data about exposure to this medication in pregnancy, and likewise towards other NSAIDs given their contraindication at specific gestation periods and the controversial findings about their reproductive safety [17,40]. Recent research has suggested a greater risk of congenital urinary tract anomalies in children prenatally exposed to nimesulide [41]. In view of this finding and of the lack of data on prenatal risks posed by nimesulide exposure, such prescribing practice should be avoided.

A key finding of this study is that 3.2% of women intentionally avoided taking a prescribed antibiotic in pregnancy, which raises concerns given the risk posed by untreated UTI and other infections on maternal-child health, including risk of pyelonephritis and premature rupture of membranes/premature delivery [42]. A study by Pisa et al. [12] in north-eastern Italy compared self-reporting with redeemed prescription data in pregnancy and showed that while 19.2% of women redeemed a systemic antibiotic prescription during gestation, only 2.6% reported their use. About 1% of women in our study also reported having intentionally avoided taking their prescribed asthma or antithrombotic medication in pregnancy. This is a somewhat unexpected finding, since heparins do not cross the placenta, and both asthma and low-dose acetylsalicylic acid have favorable safety profiles in pregnancy. Most importantly, the benefit of all these medications in pregnancy on maternal and child health outweighs by far any potential fetal risks posed by the drug itself [17]. Taken together, these findings point to the need to increase awareness among healthcare providers that a substantial number of women do not take prescribed medications at all, even when needed. Therefore, pregnant women should be given tailored, evidence-based information about both the risks and benefits of medication exposures in pregnancy so that they can be empowered to make informed treatment choices during their pregnancy. This is essential to limit unnecessary health risks for both mother and child due to inappropriate and deliberate medication non-adherence. The Teratology Information Services (TIS) available in Italy (Poison Control Center and Teratology Information Service in Bergamo, Center of perinatal toxicology in Florence, TIS in Padua and in Rome) [43] play a crucial role within this context, as they provide direct, patient-tailored advice on medication in pregnancy and breastfeeding.

Nevertheless, the identification of women who are most likely to intentionally avoid a prescribed medication in pregnancy remains challenging to date. We could not verify that key sociodemographic factors such as maternal educational level, age or occupation were independent predictors of such medication-taking behavior. We only found acute ailments and longer-term psychiatric disorders to be positively associated with the intentional avoidance of a prescribed medication. On the other hand, women who reported alcohol consumption during gestation were less likely to avoid their prescribed medications than non-consumers. Generally, alcohol use has been found to be a positive predictor of medication use in pregnancy; our study confirms this finding [10]. Because fear of harming the unborn child was one main reason for medication avoidance, future studies should elucidate whether women’s perception of risk, beliefs and knowledge about drug safety in pregnancy could be major drivers of the intentional avoidance of prescribed medication, which is necessary to ensure maternal–child health in pregnancy.

### Strengths and Limitations

A particular strength of this study is that data collection was performed over the entire country, overcoming the limitation of region-specific studies. By quantifying the extent of medication use according to self-reported indication, as short or long-term illness, it was possible to determine the leading causes for medication use among pregnant women. We corrected our prevalence estimates and association measures by survey-weighting adjustment, allowing the findings to be representative for the target population in Italy in terms of age and education. We quantified the extent and types of prescribed medications that are intentionally avoided by pregnant women, which enabled us to identify important perinatal healthcare gaps. The categorization of maternal characteristics associated with medication avoidance enabled us to identify which groups of women are more likely to need information about the benefits and risks of medication exposure during pregnancy. The utilization of an anonymous web-based questionnaire enabled us to reach a large proportion of the birthing population in Italy and limited the risk of social desirability. However, we cannot exclude the possibility that the women who decided to participate in the study differed from the general birthing population in Italy in ways for which our analysis could not control.

Several limitations need mentioning. Information about the use and avoidance of medications, as well as maternal illnesses, were based on maternal self-reporting, and thus dependent on women’s recollection and perception of illness. For new mothers, data were registered retrospectively; thus, poor recall cannot be ruled out. On the other hand, pregnant women who completed the questionnaire in early gestation may have not had the chance to develop ailments occurring in late gestation and thus utilize a medication. However, this risk of bias was carefully assessed in our prior work and considered to be minimal [10]. Because most women fear medication exposures in the first trimester due to teratogenicity risk, the above bias does not seem to be substantial for the estimates of medication avoidance. Even though clinical recommendations for the use of some medications differ according to specific periods in pregnancy, thereby influencing women’s behavior, the study did not collect data about the timing in pregnancy when a prescribed medication was purposely avoided. The questionnaire was only available through internet websites, pregnancy forums and social media; by using this kind of approach, a conventional response rate cannot be calculated and a selection bias of the target population cannot be ruled out. However, epidemiological studies have indicated the reasonable validity of web-based recruitment methods [44,45]. It has been shown that the information provided in a web-based questionnaire is equivalent in terms of quality and as reliable as that collected via traditional modes [46,47,48]. Additionally, sensitive questions can be answered more truthfully in a web-based questionnaire than in a face-to-face interview [28]. The penetration rate of the Internet either in households or at work at the time of this study was about 70% in women of childbearing age in Italy [49]. Although we made our study more generalizable in terms of age and education, selection bias due to access to the Internet cannot be excluded. Generally, our survey-weighted estimates for medication use in pregnancy did not largely deviate from unweighted estimates, although the difference in prevalence varied depending on the medication. Despite the difference in age and education that were accounted for, the women in our study more often had an occupation and were more often of Italian origin compared to the general pregnant population. Furthermore, some Italian regions were minimally represented. Lastly, we cannot rule out that unmeasured confounding by maternal beliefs and attitudes towards medications or risk perception may have influenced the magnitude of the identified associations between maternal characteristics and prescribed medication avoidance. These factors should be considered when interpreting the generalizability of the study results to the target population of Italian pregnant women.

## 5. Conclusions

In this study, we found that medication use in pregnancy, either prescribed or over-the-counter, occurs in about seven out of ten women, with no substantial differences across various geographical areas of Italy. Medications are taken mainly to treat short-term ailments, although one in every ten women uses them for the treatment of longer-term disorders. Intentional avoidance of prescribed medication is not uncommon during gestation. Avoided medications even include antibiotics, antithrombotic and asthma medications. Although medication use among pregnant women in Italy seems to be generally lower than in other Western countries, the avoidance of important medications raises concerns regarding the safeguarding of maternal–child health. To limit unnecessary and risky medication avoidance, pregnant women should be empowered to develop an evidence-based understanding not only of the potential risks but also of the benefits of medication treatments in pregnancy.

## Figures and Tables

**Table 1:** Maternal sociodemographic and health characteristics, overall and by category of medication taken in pregnancy (*n* = 926) ^1^.

Maternal Factors		Medication for Short-Term Illness		Medication for Longer-Term Illness		Medication for Both Short-and Longer-Term Illness	
	Total, *N* = 926	No, *N* = 374	Yes, *N* = 552	No, *N* = 791	Yes, *N* = 135	No, *N* = 828	Yes, *N* = 98
	*n* (%)	*n* (%)	*n* (%)	*n* (%)	*n* (%)	*n* (%)	*n* (%)
Age							
<20	11 (1.2)	4 (1.1)	7 (1.3)	10 (1.3)	<2	11 (1.3)	-
20–29	236 (25.5)	97 (25.9)	139 (25.2)	208 (26.3)	28 (20.7)	216 (26.1)	20 (20.4)
30–39	620 (67.0)	250 (66.8)	370 (67.0)	525 (66.4)	95 (70.4)	550 (66.4)	70 (71.4)
40+	59 (6.4)	23 (6.2)	36 (6.5)	48 (6.1)	11 (8.2)	51 (6.2)	8 (8.2)
Educational attainment							
Lower than high school	65 (7.0)	22 (5.9)	43 (7.8)	61 (7.7)	4 (3.0)	63 (7.6)	2 (2.0)
High school	451 (48.7)	197 (52.7)	254 (46.0)	383 (48.4)	68 (50.4)	406 (49.0)	45 (45.9)
More than high school	410 (44.3)	155 (41.4)	255 (46.2)	347 (43.9)	63 (46.7)	359 (43.4)	51 (52.0)
Marital status							
Married or cohabiting	896 (96.8)	359 (96.0)	537 (97.3)	766 (96.8)	130 (96.3)	800 (96.6)	9 (98.0)
Other than above	30 (3.2)	15 (4.0)	15 (2.7)	25 (3.2)	5 (3.7)	28 (3.4)	2 (2.0)
Occupation							
Employed	697 (75.3)	296 (79.1)	401 (72.6)	595 (75.2)	102 (75.6)	621 (75.0)	76 (77.6)
Jobless	67 (7.2)	21 (5.6)	46 (8.3)	60 (7.6)	7 (5.2)	60 (7.3)	7 (7.1)
Homemaker	83 (9.0)	28 (7.5)	55 (10.0)	68 (8.6)	15 (11.1)	73 (8.8)	10 (10.2)
Student	20 (2.2)	6 (1.6)	14 (2.5)	15 (1.9)	5 (3.7)	17 (2.1)	3 (3.1)
Other	47 (5.1)	17 (4.6)	30 (5.4)	42 (5.3)	5 (3.7)	12 (1.5)	-
No use of folic acid ^1^	29 (3.1)	11 (2.9)	18 (3.3)	23 (2.9)	6 (4.4)	24 (2.9)	5 (5.1)
No previous children	553 (59.7)	239 (63.9)	314 (56.9)	478 (60.4)	75 (55.6)	500 (60.4)	53 (54.1)
Immigrant status ^2^ (yes)	40 (4.3)	14 (3.7)	26 (4.7)	32 (4.1)	8 (5.9)	36 (4.4)	4 (4.1)
Alcohol use in pregnancy (yes) ^3^	166 (17.9)	54 (14.4)	112 (20.3)	145 (18.3)	21 (15.6)	148 (17.9)	18 (18.4)
Smoking in pregnancy (yes)	97 (10.5)	37 (9.9)	60 (10.9)	85 (10.8)	12 (8.9)	92 (11.1)	5 (5.1)
Somatic illness ^4^ (yes)	82 (8.9)	32 (8.9)	50 (9.1)	32 (4.1)	50 (37.0)	51 (6.2)	31 (31.6)
Psychiatric illness ^5^ (yes)	25 (2.7)	9 (2.4)	16 (2.9)	5 (0.6)	20 (14.8)	11 (1.3)	14 (14.3)
UTI in pregnancy	167 (18.0)	33 (8.8)	134 (24.3)	138 (17.5)	29 (21.5)	142 (17.2)	25 (25.5)
Pain in pregnancy ^6^	723 (78.1)	262 (70.1)	461 (83.5)	622 (78.6)	101 (74.8)	642 (77.5)	81 (82.7)
Nausea in pregnancy (yes)	641 (69.2)	249 (66.6)	392 (71.0)	555 (70.2)	86 (63.7)	573 (69.2)	68 (69.4)

^1^ The table shows crude, non-weighted proportions. Abbreviations: UTI = urinary tract infection. ^1^ Indicates use of folate before and/or during pregnancy. ^2^ Women having the first language different from Italian. ^3^ Indicates alcohol consumption after awareness of the pregnancy. ^4^ Includes asthma, allergy, cardiovascular and rheumatic diseases, epilepsy and diabetes type I or II. ^5^ Includes anxiety and depression. ^6^ Includes headache, pelvic or neck or back pains. Numbers may not add up to total due to missing values; missing value were 5 (0.5%) for smoking, 12 (1.3%) for alcohol use, 3 (0.3%) for immigrant status, 9 (1.0%) for folic acid use, and 12 (1.3%) for occupation.

**Table 2:** Most common medications used in pregnancy to treat short-term or longer-term illnesses (*n* = 926).

Medication (ATC Code) for Treatment of Short-Term Illness ^1^	N	Point Prevalence % (95% CI) ^2^
Paracetamol (incl. combinations) (N02BE)	411	45.3 (40.9–49.8)
Alginic acid (A02BX13)	70	9.8 (7.1–13.4)
Penicillins ± beta-lactamase inhibitors (J01CA/J01CR)	67	7.5 (5.4–10.4)
Fosfomycin (J01XX01)	37	5.0 (3.2–7.7)
Imidazole derivatives (G01AF)	30	3.6 (2.2–5.9)
Sympathomimetic nasal decongestants (R01A)	30	3.1 (1.9–5.0)
Antacids, sodium bicarbonate (A02AH)	24	2.3 (1.3–4.0)
Macrolides (J01FA)	15	1.7 (0.8–3.5)
Mucolytics (R05CB)	14	1.0 (0.6–1.8)
Antacids, magnesium compounds (A02AA)	12	1.4 (0.7–3.0)
Phloroglucinol (A03AX12)	11	2.0 (0.914.7)
Propionic acid derivates NSAID (M01AE)	10	0.7 (0.411.4)
Antacids with antiflatulents (A02AF)	9	0.6 (0.3–1.2)
Proton pump inhibitors (A02BC)	8	0.6 (0.3–1.3)
Acetylsalicylic acid (incl. combinations) (N02BA)	8	0.5 (0.3–1.1)
Third-generation cephalosporins (J01DD)	5	0.4 (0.2–1.1)
Nimesulide (M01AX17)	5	0.3 (0.1–0.9)
Anxiolytics, benzodiazepine (N05BA)	5	0.4 (0.2–1.0)
Selective serotonin (5-HT1) agonists (N02CC)	5	1.3 (0.4–4.1)
**Medication (ATC Code) for Treatment of Longer-Term Illness ^1^**		
Levothyroxine (H03AA01)	59	5.1 (3.7–6.9)
Anxiolytics, benzodiazepine (N05BA)	11	1.4 (0.7–3.0)
Inhalant selective beta-2 agonists (R03AC)	11	1.4 (0.6–3.2)
Heparins (B01AB)	11	0.9 (0.5–1.6)
Low dose acetylsalicylic acid (B01AC)	11	0.9 (0.5–1.6)
SSRI antidepressants (N06AB)	9	1.1 (0.4–2.7)
Systemic glucocorticoids (H02AB)	4	0.8 (0.2–3.1)
Antiepileptics Antiepileptics (N03A)	4	0.3 (0.1–0.9)
Adrenergics and other drugs for COPD (R03AK)	4	0.4 (0.1–1.0)
Inhaled glucocorticoids (R03BA)	4	0.4 (0.1–1.1)

^1^ Medications taken by <4 women are not shown. ^2^ Weighted proportion, by survey weighting. Magnitude of proportions may not directly align with number of women in the various categories because proportions are weighted. Abbreviations: ATC = Anatomical Therapeutic Chemical; 95% CI: 95% confidence interval; NSAID = Non-steroidal anti-inflammatory drugs; SSRI = selective serotonin re-uptake inhibitors; COPD = chronic obstructive pulmonary disease.

**Table 3:** Adjusted odds ratio (OR) for intentional avoidance of prescribed medication in pregnancy ^1^.

Maternal Factor	Adjusted OR (95% CI)	*p*-Value
**Alcohol Use During Pregnancy ^2^**		
No	1.0 (Reference)	
Yes	0.58 (0.36–0.94)	0.028
**Nausea In Pregnancy**		
No	1.0 (Reference)	
Yes	1.55 (0.99–2.43)	0.055
**Urinary Tract Infection in Pregnancy**		
No	1.0 (Reference)	
Yes	1.76 (1.04–2.96)	0.034
**Long-Term Psychiatric Illness in Pregnancy**		
No	1.0 (Reference)	
Yes	2.73 (0.95–7.88)	0.063

^1^ The maternal factors showed in the table were the only significant independent variables retained in the final multivariate model. ^2^ Indicates alcohol use after awareness of the pregnancy.

## Supplementary Materials

The following are available online at https://www.mdpi.com/1660-4601/17/11/3830/s1, Figure S1: Number of responses to the study electronic questionnaires across the regions in Italy, Figure S2: Extent of medication used in pregnancy, overall and by treatment of short-term or longer-term illnesses, accordingly to geographical areas (*n* = 926), Table S1: Distribution of key indicators of our population versus the birthing population in Italy in 2012 based on CEDAP data, Table S2: Overall medication use on 3rd with/without 4th ATC level, by trimester of use (*n* = 9459).
