# Supplementary material for: Use and Intentional Avoidance of Prescribed Medications in Pregnancy: A Cross-Sectional, Web-Based Study among 926 Women in Italy
Source: Int J Environ Res Public Health. 2020 May 28;17(11):3830. doi: 10.3390/ijerph17113830 (PMC7312729; doi:10.3390/ijerph17113830)
Supplement: Supplementary file 1 [file ijerph-17-03830-s001.pdf]

Supplementary material

Figure S1: Number of responses to the study electronic questionnaires across the regions in Italy

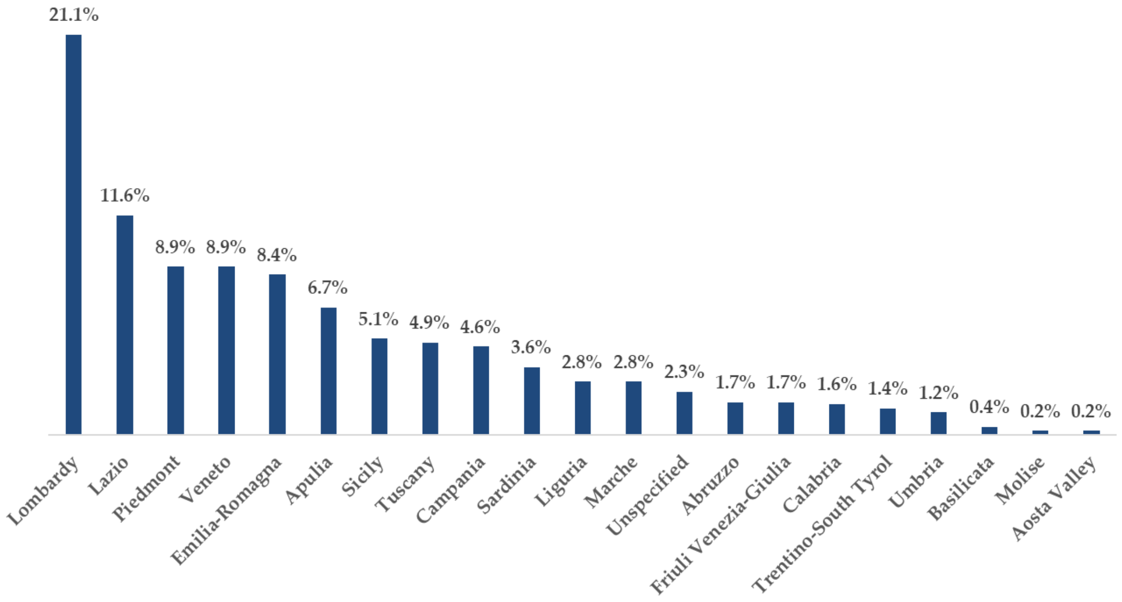

Figure S2: Extent of medication used in pregnancy, overall and by treatment of short-term or longer-term illnesses, accordingly to geographical areas (n=926)[1]

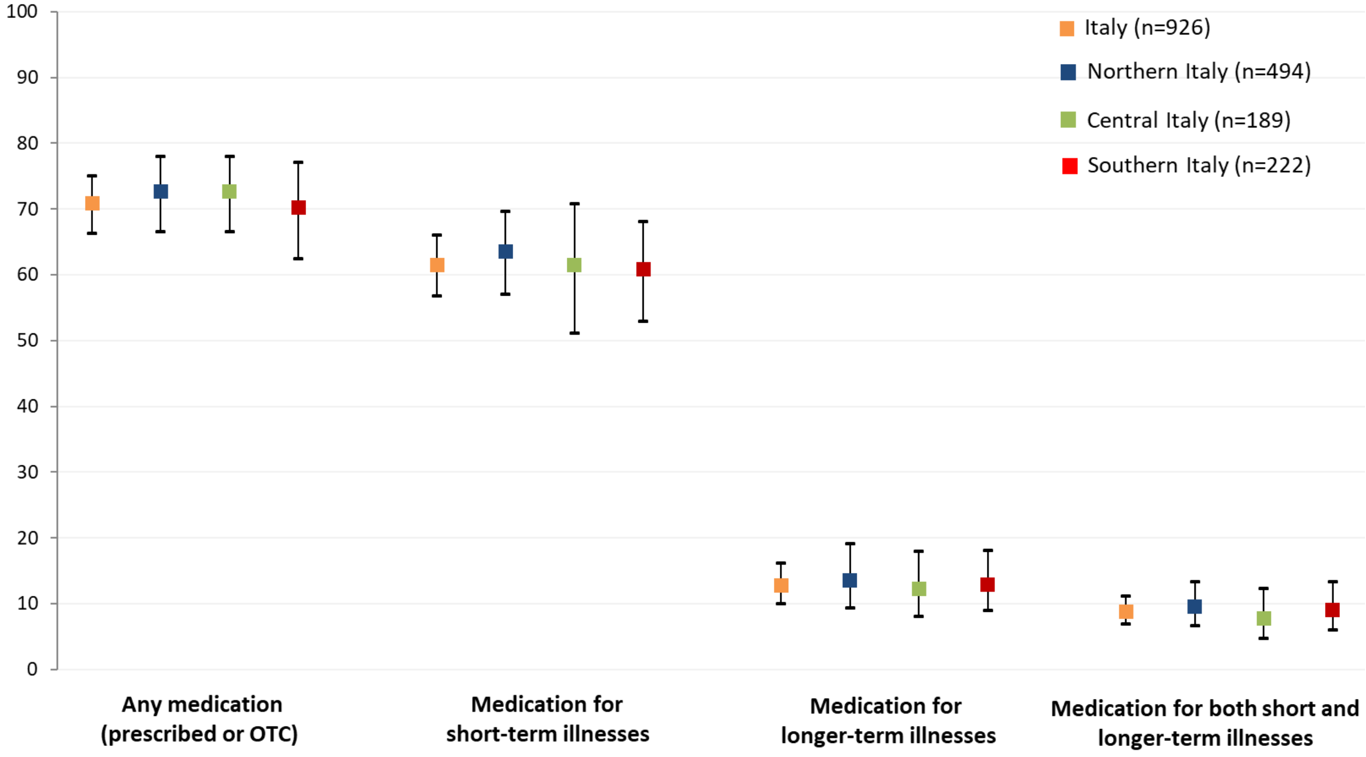

[1]Region of residency was missing for 21 women.
Northern Italy includes Lombardy, Piedmont, Trentino-South Tyrol, Aosta Valley, Veneto, Emilia-Romagna, Liguria, Friuli Venezia-Giulia. Central Italy includes Lazio, Marche, Tuscany, Umbria. Southern Italy includes Sicily, Sardinia, Apulia, Molise, Calabria, Basilicata, and Abruzzo.

Table S1: Distribution of key indicators of our population versus the birthing population in Italy in 2012 based on CEDAP data[1]

Maternal factors	Study population %	Birthing population %
**Age**		
< 20	1.2	1.5
20-29	25.5	30.3
30-39	67.0	59.7
40+	6.4	8.5
**Educational attainment**		
Lower than high school	7.0	30.6
High school	48.7	44.3
More than high school	44.3	25.1
**Marital status**		
Married or cohabiting	96.8	97.4
Other than above	3.2	2.6
**Occupation**		
Working	75.3	58.4
Jobless	7.2	9.8
Homemaker	9.0	29.8
Student	2.2	1.6
Other	5.1	0.4
**Immigrant status**		
No	95.7	80.1
Yes	4.3	19.9

1. *Ministry of Health. Certificate of Delivery Assistance (CEDAP)*. 2012 [cited 2020 1 March]; Available from: http://www.salute.gov.it/portale/documentazione/p6_2_2_1.jsp?lingua=italiano&id=2768.

Table S2: Overall medication use on 3rd with/without 4th ATC level, by trimester of use (n=9,459)[1]

Medication by pharmacological subgroup (ATC at 3rd level), with or without 4th level chemical subgroup		Weighted proportions 1st trimester n % (95% CI)	Weighted proportions 2nd trimester n % (95% CI)	Weighted proportions 3rd trimester n % (95% CI)	Unweighted proportions 1st trimester n % (95% CI)	Unweighted proportions 2nd trimester n % (95% CI)	Unweighted proportions 3rd trimester n % (95% CI)
**A02A**	Antacids	57<br>6.4 (4.5-9.0)	68<br>7.5 (5.4-10.2)	75<br>9.0 (6.6-12.1)	57<br>6.2 (4.8-7.9)	68<br>7.3 (5.8-9.2)	75<br>8.1 (6.5-10.0)
**A02AA**	Antacids, magnesium compounds	10<br>1.2 (0.5-2.8)	5<br>0.8 (0.3-2.6)	<4	10<br>1.1 (0.6-2.0)	5<br>0.5 (0.2-1.3)	<4
**A02AD**	Antacids, aluminium, calcium and magnesium combinations	38<br>4.5 (1.0-6.9)	52<br>5.9 (1.1-8.5)	57<br>7.6 (1.4-10.8)	38<br>4.1 (3.0-5.6)	52<br>5.6 (4.3-7.3)	57<br>6.2 (4.8-7.9)
**A02AF**	Antacids with antiflatulents	<4	5<br>0.4 (0.2-0.9)	6<br>0.4 (0.2-1.0)	<4	5<br>0.5 (0.2-1.3)	6<br>0.6 (0.3-1.4)
**A02AH**	Antacids, sodium bicarbonate	12<br>1.4 (0.6-3.2)	10<br>1.2 (0.6-3.0)	15<br>1.6 (0.6-3.4)	12<br>1.3 (0.7-2.3)	10<br>1.1 (0.6-2.0)	15<br>1.6 (1.0-2.7)
**A02B**	Drugs for peptic ulcer and gastro-esophageal reflux disease	29<br>4.4 (2.6-7.2)	42<br>5.9 (3.9-8.8)	38<br>4.7 (3.0-7.2)	29<br>3.1 (2.2-4.5)	42<br>4.5 (3.4-6.1)	38<br>4.1 (3.0-5.6)
**A02BC**	Proton pump inhibitors	5<br>0.4 (0.2-0.9)	4<br>0.3 (0.1-0.7)	5<br>0.4 (0.2-0.9)	5<br>0.5 (0.2-1.3)	4<br>0.4 (0.2-1.1)	5<br>0.5 (0.2-1.3)
**A02BX**	Other drugs for peptic ulcer and gastro-esophageal reflux disease, mainly alginic acid	24<br>4.0 (2.3-6.9)	38<br>5.6 (3.6-8.6)	35<br>4.5 (2.8-7.0)	24<br>2.6 (1.7-3.8)	38<br>4.1 (3.0-5.6)	35<br>3.8 (2.7-5.2)
**A03A**	Drugs for functional gastrointestinal disorders	10<br>2.0 (0.8-4.7)	5<br>0.7 (0.2-2.6)	<4	10<br>1.1 (0.6-2.0)	5<br>0.5 (0.2-1.2)	<4
**A03AX**	Other drugs for functional gastrointestinal disorders, mainly phloroglucinol,	8<br>1.8 (0.7-4.7)	4<br>0.6 (0.2-2.6)	<4	8<br>0.9 (0.4-1.7)	4<br>0.4 (0.2-1.1)	<4
**A03B**	Belladonna alkaloids and derivatives	17<br>2.3 (1.1-4.5)	21<br>3.0 (1.6-5.5)	11<br>1.4 (0.6-3.2)	17<br>1.8 (1.1-2.9)	21<br>2.3 (1.5-3.5)	11<br>1.2 (0.7-2.1)
**A03BB**	Butylscopolamine	17<br>2.3 (1.1-4.5)	21<br>3.0 (1.6-5.5)	11<br>1.4 (0.6-3.2)	17<br>1.8 (1.1-2.9)	21<br>2.3 (1.4-3.5)	11<br>1.2 (0.7-2.1)

Medication by pharmacological subgroup (ATC at 3rd level), with or without 4th level chemical subgroup		Weighted proportions			Unweighted proportions		
		1st trimester n % (95% CI)	2nd trimester n % (95% CI)	3rd trimester n % (95% CI)	1st trimester n % (95% CI)	2nd trimester n % (95% CI)	3rd trimester n % (95% CI)
**A03F**	Propulsives	25<br>3.0 (1.7-5.2)	10<br>0.8 (0.4-1.5)	<4	25<br>2.7 (1.8-4.0)	10<br>1.1 (0.6-2.0)	<4
**A03FA**	Metoclopramide or domperidone	25<br>3.0 (1.7-5.2)	10<br>0.8 (0.4-1.5)	<4	25<br>2.7 (1.8-4.0)	10<br>1.1 (0.6-2.0)	<4
**A06A**	Drugs for constipation	34<br>3.7 (2.3-5.7)	52<br>5.3 (3.8-7.5)	36<br>3.1 (2.2-4.3)	34<br>3.7 (2.6-5.1)	52<br>5.6 (4.3-7.3)	36<br>3.9 (2.8-5.3)
**A06AB**	Contact laxatives	4<br>0.4 (0.1-1.0)	5<br>0.5 (0.2-1.1)	<4	4<br>0.4 (0.2-1.1)	5<br>0.5 (0.2-1.3)	<4
**A06AD**	Osmotically acting laxatives	16<br>1.2 (0.7-2.1)	31<br>3.1 (2.0-4.9)	19<br>1.7 (1.1-2.6)	16<br>1.7 (1.1-2.8)	31<br>3.3 (2.4-4.7)	19<br>2.1 (1.3-3.2)
**A06AG**	Enemas	5<br>0.4 (0.2-0.9)	4<br>0.3 (0.1-0.9)	4<br>0.4 (0.1-1.0)	5<br>0.5 (0.2-1.3)	4<br>0.4 (0.2-1.1)	4<br>0.4 (0.2-1.1)
**A06AX**	Other drugs for constipation (glycerol)	13<br>1.9 (0.9-4.1)	15<br>1.6 (0.8-3.1)	9<br>0.8 (0.4-1.5)	13<br>1.4 (0.8-2.4)	15<br>1.6 (1.0-2.7)	9<br>1.0 (0.5-1.9)
**B01A**	Antithrombotic agents	13<br>1.0 (0.6-1.8)	17<br>1.4 (0.8-2.2)	13<br>1.0 (0.6-1.7)	13<br>1.4 (0.8-2.4)	17<br>1.8 (1.1-2.9)	13<br>1.4 (0.8-2.4)
**B01AB**	Heparins	10<br>0.8 (0.4-1.4)	10<br>0.8 (0.4-1.5)	7<br>0.5 (0.2-1.2)	10<br>1.0 (0.6-2.0)	10<br>1.1 (0.6-2.0)	7<br>0.8 (0.4-1.6)
**B01AC**	Low dose acetylsalicylic acid	7<br>0.6 (0.3-1.2)	10<br>0.8 (0.4-1.5)	9<br>0.7 (0.3-1.3)	7<br>0.8 (0.4-1.6)	10<br>1.1 (0.6-2.0)	9<br>1.0 (0.5-1.9)
**G01A**	Anti-infective and antiseptic drugs, excluding combinations with corticosteroids	10<br>0.8 (0.4-1.5)	22<br>2.5 (1.4-4.4)	17<br>2.2 (1.1-4.3)	10<br>1.1 (0.6-2.0)	22<br>2.4 (1.6-3.6)	17<br>1.8 (1.1-2.9)
**G01AA**	Antibiotics	<4	4<br>0.3 (0.1-0.8)	<4	<4	4<br>0.4 (0.2-1.1)	<4
**G01AF**	Imidazole derivatives	9<br>0.7 (0.4-1.4)	16<br>2.0 (1.0-4.0)	10<br>1.2 (0.5-3.1)	9<br>1.0 (0.5-1.9)	16<br>1.7 (1.1-2.8)	10<br>1.1 (0.6-2.0)
**H02A**	Systemic corticosteroids	4<br>0.4 (0.1-1.0)	4<br>0.8 (0.2-3.1)	4<br>0.8 (0.2-3.1)	4<br>0.4 (0.2-1.1)	4<br>0.4 (0.2-1.1)	4<br>0.4 (0.2-1.1)

Medication by pharmacological subgroup (ATC at 3rd level), with or without 4th level chemical subgroup		Weighted proportions			Unweighted proportions		
		1st trimester<br>n<br>% (95% CI)	2nd trimester<br>n<br>% (95% CI)	3rd trimester<br>n<br>% (95% CI)	1st trimester<br>n<br>% (95% CI)	2nd trimester<br>n<br>% (95% CI)	3rd trimester<br>n<br>% (95% CI)
**H03A**	Thyroid preparations	53<br>4.6 (3.3-6.4)	43<br>3.8 (2.6-5.6)	37<br>3.3 (2.1-5.0)	53<br>5.7 (4.4-7.4)	43<br>4.6 (3.5-6.2)	37<br>4.0 (2.9-5.5)
**H03AA**	Thyroid hormone, mainly levothyroxine	53<br>4.6 (3.3-6.4)	43<br>3.8 (2.6-5.6)	37<br>3.3 (2.1-5.0)	53<br>5.7 (4.4-7.4)	43<br>4.6 (3.5-6.2)	37<br>4.0 (2.9-5.5)
**J01C**	Penicillins	16<br>1.3 (0.8-2.1)	36<br>4.6 (2.9-7.3)	26<br>2.9 (1.7-5.0)	16<br>1.7 (1.1-2.8)	36<br>3.9 (2.8-5.3)	26<br>2.9 (1.9-4.1)
**J01CA**	Penicillins with extended spectrum	11<br>0.9 (0.5-1.6)	21<br>2.6 (1.4-4.8)	12<br>1.8 (0.8-4.0)	11<br>1.2 (0.7-2.1)	21<br>2.3 (1.5-3.5)	12<br>1.3 (0.7-2.3)
**J01CR**	Penicillins and beta-lactamase inhibitors	5<br>0.4 (0.2-1.0)	14<br>2.5 (1.2-5.1)	14<br>1.5 (0.8-3.1)	5<br>0.5 (0.2-1.3)	14<br>1.5 (0.9-2.5))	14<br>1.5 (0.9-2.5)
**J01D**	Other beta-lactam antibacterials (mainly third-generation cephalosporins)	-	<4	4<br>0.4 (0.1-1.0)	-	<4	4<br>0.4 (0.2-1.1)
**J01F**	Macrolides	<4	7<br>1.1 (0.4-3.1)	10<br>0.8 (0.4-1.5)	<4	7<br>0.8 (0.4-1.6)	10<br>1.1 (0.6-2.0)
**J01X**	Other anti-bacterials	16<br>1.6 (0.8-3.1)	19<br>2.3 (1.2-4.2)	14<br>2.0 (0.9-4.1)	16<br>1.7 (1.1-2.8)	19<br>2.1 (1.3-3.2)	14<br>1.5 (0.9-2.5)
**J01XX**	Other anti-bacterials, mainly fosfomycin	15<br>1.5 (0.7-3.0)	19<br>2.3 (1.2-4.2)	13<br>1.9 (0.8-4.1)	15<br>1.6 (1.0-2.7)	19<br>2.1 (1.3-3.2)	13<br>1.4 (0.8-2.4)
**M01A**	NSAID preparations	14<br>1.0 (0.6-1.7)	7<br>0.6 (0.3-1.2)	5<br>0.3 (0.1-0.8)	14<br>1.5 (0.9-2.5)	7<br>0.8 (0.4-1.6)	5<br>0.5 (0.2-1.3)
**M01AE**	Propionic acid derivates	7<br>0.5 (0.2-1.1)	<4	<4	7<br>0.8 (0.4-1.6)	<4	<4
**M01AX**	Other NSAIDs, mainly nimesulide	4<br>0.3 (0.1-0.8)	<4	<4	4<br>0.4 (0.2-1.1)	<4	<4
**N02B**	Analgesics and antipyretics	206<br>23.5 (19.7-27.6)	258<br>30.7 (26.6-35.2)	145<br>17.5 (14.1-21.5)	206<br>22.2 (19.7-25.0)	258<br>27.9 (25.1-30.8)	145<br>15.7 (13.5-18.1)

Medication by pharmacological subgroup (ATC at 3rd level), with or without 4th level chemical subgroup		Weighted proportions 1st trimester n % (95% CI)	Weighted proportions 2nd trimester n % (95% CI)	Weighted proportions 3rd trimester n % (95% CI)	Unweighted proportions 1st trimester n % (95% CI)	Unweighted proportions 2nd trimester n % (95% CI)	Unweighted proportions 3rd trimester n % (95% CI)
**N02BA**	Salicylic acid and derivatives, mainly high dose acetylsalicylic acid	5<br>0.3 (0.1-0.8)	<4	<4	5<br>0.5 (0.2-1.3)	<4	<4
**N02BE**	Anilides, mainly paracetamol	202<br>23.2 (19.5-27.4)	191<br>24.1 (21.2-28.5)	144<br>17.4 (14.1-21.3)	202<br>21.8 (19.3-24.6)	191<br>20.6 (18.1-23.4)	144<br>15.6 (13.4-18.0)
**N02C**	Antimigraine drugs	5<br>1.3 (0.4-4.1)	5<br>1.3 (0.4-4.1)	<4	5<br>0.5 (0.2-1.3)	5<br>0.5 (0.2-1.3)	<4
**N05B**	Anxiolytics	11<br>1.3 (0.6-2.9)	9<br>0.8 (0.4-1.5)	4<br>0.4 (0.1-1.0)	11<br>1.2 (0.7-2.1)	9<br>1.0 (0.5-1.9)	4<br>0.4 (0.2-1.1)
**N05BA**	Benzodiazepine derivatives	10<br>1.3 (0.6-2.9)	9<br>0.8 (0.4-1.5)	4<br>0.4 (0.1-1.0)	10<br>1.1 (0.6-2.0)	9<br>1.0 (0.5-1.9)	4<br>0.4 (0.2-1.1)
**N06A**	Antidepressants	11<br>1.3 (0.6-2.9)	10<br>0.8 (0.4-1.5)	9<br>0.7 (0.3-1.3)	11<br>1.2 (0.7-2.1)	10<br>1.1 (0.6-2.0)	9<br>1.0 (0.5-1.9)
**N06AB**	SSRI antidepressants	8<br>1.0 (0.4-2.7)	6<br>0.4 (0.2-1.0)	5<br>0.3 (0.1-0.8)	8<br>0.9 (0.4-1.7)	6<br>0.6 (0.3-1.4)	5<br>0.5 (0.2-1.3)
**R01A**	Sympathomimetic nasal decongestants	12<br>1.0 (0.5-1.7)	17<br>1.3 (0.8-2.1)	11<br>1.5 (0.6-3.6)	12<br>1.3 (0.7-2.3)	17<br>1.8 (1.1-2.9)	11<br>1.2 (0.7-2.1)
**R01AA**	Plain sympathomimetic nasal decongestants	8<br>0.6 (0.3-1.3)	11<br>0.8 (0.4-1.5)	6<br>1.2 (0.4-3.5)	8<br>0.9 (0.4-1.7)	11<br>1.2 (0.7-2.1)	6<br>0.6 (0.3-1.4)
**R01AB**	Combination of sympathomimetic nasal decongestants, excluding corticosteroids	<4	6<br>0.5 (0.2-1.2)	<4	<4	6<br>0.6 (0.3-1.4)	<4
**R01AD**	Corticosteroids	<4	4<br>0.3 (0.1-0.8)	<4	<4	4<br>0.4 (0.2-1.1)	<4
**R03A**	Inhalant adrenergics	9<br>0.9 (0.4-1.7)	8<br>1.2 (0.5-3.1)	7<br>1.0 (0.3-3.0)	9<br>1.0 (0.5-1.9)	8<br>0.9 (0.4-1.7)	7<br>0.8 (0.4-1.6)
**R03AC**	Inhalant selective beta-2 agonists	6<br>0.6 (0.3-1.3)	7<br>1.1 (0.4-3.1)	5<br>0.8 (0.2-3.0)	6<br>0.6 (0.3-1.4)	7<br>0.8 (0.4-1.6)	5<br>0.5 (0.2-1.3)

Medication by pharmacological subgroup (ATC at 3rd level), with or without 4th level chemical subgroup		Weighted proportions			Unweighted proportions		
		1st trimester<br>n<br>% (95% CI)	2nd trimester<br>n<br>% (95% CI)	3rd trimester<br>n<br>% (95% CI)	1st trimester<br>n<br>% (95% CI)	2nd trimester<br>n<br>% (95% CI)	3rd trimester<br>n<br>% (95% CI)
**R03B**	Other inhalant drugs for COPD	4<br>0.3 (0.1-0.9)	6<br>0.6 (0.2-1.2)	4<br>0.3 (0.1-0.9)	4<br>0.4 (0.2-1.1)	6<br>0.6 (0.3-1.4)	4<br>0.4 (0.2-1.1)
**R03BA**	Glucocorticoids	4<br>0.3 (0.1-0.9)	6<br>0.6 (0.2-1.2)	4<br>0.3 (0.1-0.9)	4<br>0.4 (0.2-1.1)	6<br>0.6 (0.3-1.4)	4<br>0.4 (0.2-1.1)
**R05C**	Expectorants	<4	10<br>0.7 (0.4-1.3)	5<br>0.4 (0.2-1.0)	<4	10<br>1.1 (0.6-2.0)	5<br>0.4 (0.2-1.3)
**R05CB**	Mucolytics, mainly acetylcysteine	<4	10<br>0.7 (0.4-1.3)	5<br>0.4 (0.2-1.0)	<4	10<br>1.1 (0.6-2.0)	5<br>0.5 (0.2-1.3)
**R06A**	Systemic antihistamines	<4	4<br>0.3 (0.1-0.9)	<4	<4	4<br>0.4 (0.2-1.1)	<4
**Total medication use (any ATC)**		**394<br>43.9 (39.5-48.4)**	**411<br>47.1 (42.7-51.6)**	**289<br>31.1 (27.1-35.4)**	**394<br>42.5 (39.4-45.8)**	**411<br>44.4 (41.2-47.7)**	**289<br>31.2 (28.3-34.3)**

Abbreviations: NSAID=Non-steroidal anti-inflammatory drugs; SSRI=Selective serotonin re-uptake inhibitors; COPD=Chronic obstructive pulmonary disease.

[1]Only medication groups with at least 4 exposed pregnancies in one of the trimester are presented. Numbers less than 4 are presented as "<4". Point estimate by 4th ATC level are presented only when there are at least 6 exposed to the 3rd level ATC medication group. Timing of medication exposure is defined as follows: 1st trimester (gestational weeks 0-12), 2nd trimester (gestational week 13-24), 3rd trimester (gestational week 25 and up to childbirth).
